# Why are some people in the UK reluctant to seek support for their pets?

**DOI:** 10.1017/awf.2024.19

**Published:** 2024-05-03

**Authors:** Janine C Muldoon, Joanne M Williams

**Affiliations:** Children, adolescents and animals research (Caar), Clinical and Health Psychology, University of Edinburgh, Doorway 6, Medical School, Elsie Inglis Quad, Teviot Place, Edinburgh EH8 9AG, UK

**Keywords:** animal welfare, challenges, companion animals, narrative research, stigma, support-seeking

## Abstract

Drawing upon data from a study examining experiences of accessing support for pets from the UK animal welfare charity Blue Cross, this paper illuminates reasons why people might not seek support when they need it. This applies to those who are struggling financially and are eligible for, but do not take, free/reduced cost veterinary care, or are having other problems (e.g. the animal’s disruptive behaviour or ill health, struggling to care for the pet due to changing circumstances or health problems, or coping with pet loss). Twenty Blue Cross service users (15 female, five male, age 29–67) took part in individual online interviews using a semi-guided narrative approach, where they were encouraged to share their experiences of reaching out. They were also asked to reflect upon why others may not do the same, and if they had any recommendations for organisations to help them reach these people. Findings echo other studies that highlight a fear of being judged, disclosure and stigma. Guilt, shame, lack of awareness, financial concerns, and wanting to manage independently, all play important roles. These factors have implications for the way support services are advertised and delivered to ensure animals receive the care needed. We describe these reflections and recommendations and identify three broader ideological narratives underpinning participants’ stories: ‘giving back’; ‘equity’, and ‘sacrifice’. These reveal how wider societal attitudes and values shape identities and behaviours. It is vital that support on offer is reframed to explicitly counteract these influences to ensure optimal animal and human welfare.

## Introduction

Animal welfare charities know that they are not reaching every pet owner who needs their support (Blue Cross 2022, personal communication 2023; Royal Society for the Protection of Animals [RSPCA] 2023; Scottish SPCA 2023) and believe that we are in the midst of an ‘animal welfare crisis’. Demand for financial assistance with pet care is at an all-time high, while charities are experiencing challenges due to staff shortages, supply problems, and increasing costs. Newspaper headlines abound with news of shelters “drowning” in animals (Murray 2022), “soaring numbers of abandoned puppies” (Duffin 2023), and pet owners being urged to use pet food banks due to “huge surge in abandoned cats and dogs” (Dalton 2023). These articles lay bare that there are now far more animals needing help than animal charities can cope well with, and fewer people adopting (Dogs’ Trust 2023a; RSPCA *et al.* 2023), so animals stay longer in charity care before someone comes forward. While the cost-of-living crisis in the UK has led more people to seek support to help them care for their pet, it is clear that many others have instead been driven to relinquish them.

There has been a steadily increasing rise in the number of animals being abandoned in the UK since the COVID-19 pandemic (RSPCA 2023). Compared with the same period the previous year, there was an 11% increase in 2023, and a 72% increase in the number of people clicking on the RSPCA ‘giving up a pet’ webpage (RSPCA *et al*. 2023). Similarly, the Scottish SPCA (2023) notes an 11% increase in calls to give up animals, and a 27% increase in animals coming into their care. The Dogs’ Trust (2023b) has also seen a record high in the number of people contacting them because they need to give up their dogs. Animal welfare charities are keen to support as many people as they can, but this requires pet owners to come forward before the situation becomes untenable and there is no other option than to give them up. Research on pet relinquishment has shown that this is not done lightly or without emotion. There is a process or struggle that individuals undergo prior to making this decision (DiGiacomo et al. 1998). At present, however, there has been no research shedding light upon why people might not seek help when they are struggling to care for their pets in the first instance. We need to turn to other areas of research to illuminate the root causes. One such area is non-take-up of means-tested benefits.

At the start of the COVID-19 pandemic, it is estimated that around half a million people in the UK who were eligible for Universal Credit (UC; Gov.uk 2023a) did not claim it (Baumberg et al. 2021). Approximately 50% of these people thought they would be eligible but did not want to claim (a third saying they did not need it). However, the most common reason for not applying was the perceived hassle – the challenge of working out if they were eligible, the claims process itself (see Cheetham et al. 2019; p 1 who describe it as “impersonal, hostile and demeaning”), or the threat of sanctions, alternatively known as the “costs of compliance” (Bennett et al. 2009). A further 27% did not claim UC because of stigma – the association of benefits with a “devalued social identity” (Baumberg 2016). Baumberg et al. (2021) also found that up to 390,000 people wrongly believed they were ineligible. This highlights that people are not always aware there is support available for them (LaVallee et al. 2017). They may assume services are only available to those in a far worse situation (see Craig 1991; Fong et al. 2016). Both groups are of concern though, because when asked specific questions, it was clear they were experiencing significant financial strain (e.g. not being able to afford an unanticipated exceptional cost such as replacing or repairing a fridge, and not being able to afford fresh fruit and vegetables daily). They also had worse mental health than the general public (excluding claimants). People tend to delay claiming as long as possible, waiting instead to see if things improve and viewing others as more in need than them (Shildrick & MacDonald 2013). It is often the last resort after they have tried everything else.

We know that people worry about the responsibility they have for their pets, and feel guilty, angry and helpless if they are unable to provide for their needs (Merkouri et al. 2022). They often prioritise the pet above themselves (Rauktis et al. 2017; Friedman *et al.* 2020), particularly where food is concerned. Worrying about “food precarity” is linked to feelings of confusion, frustration, anxiety, and shame (Ivancic & Dooling 2023; p 294). Therefore, the stigma and conditionality that have long been recognised as barriers to applying for means-tested benefits or charitable support (Shildrick & MacDonald 2013; Baumberg et al. 2021; Marc *et al*. 2022; Inglis et al. 2023) undoubtedly apply where pets are concerned. The likelihood of seeking support to meet someone else’s needs may be increased, but it may still be associated with the same anguish. Baumberg et al. (2021) distinguish between three different types of stigma relating to claiming benefits that might equally apply to poverty in general: ‘personal stigma’: a person’s own feeling that those claiming benefits/experiencing poverty should be looked down upon; ‘stigmatisation’: the perception that others look down upon those claiming benefits/in poverty, and ‘claims stigma’: the feeling of being looked down upon while claiming benefits/seeking support (e.g. respect and privacy shown by Jobcentre/claims staff).

The avoidance of stigma is understandable. Experiences of poverty stigma have been found to be associated with negative self-evaluations, diminished social well-being, negative affect, and mental ill-health (Hill & Webber 2022; Inglis et al. 2023). However, there is perhaps also a broader stigma associated with seeking help of any kind. In their study of students in UK higher education, Clegg et al. (2006) found that most associated seeking help with failure or loss of face. Tending to accept that life can be difficult, they did not view their difficulties as something that should prompt them to seek help. Similar findings emerge from studies exploring avoidance of help-seeking for mental health problems (Clement et al. 2015). Such problems are often normalised, their seriousness downplayed, and people want to manage the problem themselves. Poor knowledge of services, concerns about confidentiality and disclosure, and fear or stress about the process of seeking help, are key barriers. Clement et al. (2015; p 21) conclude that it is important to view stigma “as part of a larger network of beliefs and other constraints deterring help-seeking”.

This paper is one in a series of publications that focus on findings from an in-depth qualitative study examining pet owners’ experiences of accessing support for pets from the long-established UK animal welfare charity Blue Cross. This accompanied an annual online survey in 2022 and 2023 (see Muldoon & Williams 2024). The study also examined the challenges associated with pet ownership (Muldoon *et al.* under review), and the impact of financial hardship on pet owners (Muldoon & Williams, in press). Exploring the potential reasons behind a reluctance to seek support, we examine:

- the experience of initially accessing support (what facilitates reaching out and what problems are encountered?);
- participants’ reflections on what might stop someone reaching out for support in the way they have done;
- their suggestions on how organisations might improve their services in order to reach these people; and
- wider narratives at play that shed light upon how societal attitudes and values might be implicated in a reluctance to seek support for pets.

## Materials and methods

### Participants

The sample included 15 female and five male English-speaking participants aged 29–67 years (mean [± SD] = 51.35 [± 10.96]). Fifteen were registered with the veterinary hospitals (VH), seven using the Grimsby VH in Northeast Lincolnshire and eight the London VHs (in Victoria and Hammersmith). Ten people had only accessed a VH, two females had also used the pet bereavement support service, one female the behavioural support service, and a male/female couple a pet food bank. Of the remaining five (all female from southern England or the Midlands), four were not experiencing financial hardship. Three had just used the behavioural support service, two for a dog and cat rehomed from Blue Cross, and one for a dog bought from a breeder. One female participant had just used a pet food bank. Participants mostly discussed accessing services for their current pets, but several shared experiences with pets who had now passed away. Seventeen participants had dogs (now or in the past), seven cats, and two ponies.

### Procedure

From November 2022 to April 2023, 20 Blue Cross service users took part in an online interview with the lead author of the paper (JM). Participants were recruited in stages to avoid too many clients volunteering to take part. An email from the research team with an information sheet and link to an online consent form in Qualtrics was forwarded by Blue Cross to clients who had given their permission to receive emails for marketing or research purposes. They were advised of the £20 Amazon voucher they would receive for taking part. Blue Cross sent out small batches of emails to hospital clients in London and Grimsby and those who had used the behavioural or rehoming services. Staff at pet food banks were also asked to promote the study to visiting clients.

Once those wishing to participate had completed the consent form, the researcher organised a date and time to meet. Interviews took approximately 40 min and were carried out in Microsoft® Teams and audio-recorded using Online Broadcasting Software (OBS). The transcribe function in Word was used to generate an initial transcript, which was then reviewed and amended. No names/identifying information were included. Pseudonyms were created for people and pets. Participants were sent a thank you email with contact details for support organisations and a link to our webpage to access the summary of the findings.

### Interview methodology

Individual semi-guided interviews were used to explore each participant’s journey of care with Blue Cross – from the experiences that initially lead them to reach out for support and the extent of the challenges experienced with pets, through to how the care received has impacted them and their pet. They were also asked, at particular times, to reflect upon why others who find themselves in a similar (or worse) situation might not seek support. A narrative approach was used in the interviews (Butina 2015; Wong & Breheny 2018, 2021). This foregrounds participants’ stories, with the researcher providing the opportunity for people to narrate their experiences instead of using a strict question-answer format. However, in recognition that some people find it difficult to tell their story in this way (Anderson & Kirkpatrick 2016), a semi-structured method was used, following a set of guiding questions/themes to help capture the chronology of events (see Table S1 in the Supplementary material). The structure was not followed rigidly or linearly. Space was left for participants to determine the direction of the discussion.

#### The narrative approach

Narrative psychology refers to the ways people construct a story of self and experience to satisfy the need for coherence and meaning. While people obviously tell stories that are personal to them, they are deeply socially and culturally embedded (Riessman 2008). They are built on “taken for granted understandings of how social life does and should work. Because of this, the stories people tell give us insights into the, often unspoken, rules for reacting to and interacting with the world, because they reflect broader narratives of social life that we have absorbed” (Wong & Breheny 2018; p 246). Broader cultural narratives, often termed “ideological” (Haidt et al. 2009) or “positional” (Murray 2000), are drawn on to tell personal stories (accounts of events), allowing people to actively position their identity relative to others, as they explain their actions and justify ways in which they behave (Skultans 2000).

### Data analysis

The analysis for this paper focused on identifying potential reasons that could explain why some people are reluctant to seek support for a pet when they are struggling, financially or otherwise. Initially, JM adopted an in-depth, case-driven inductive approach to analysing all the data, with an eye to broader narratives at play (reflecting wider societal norms, attitudes or values). Anonymised transcripts were imported into NVivo and analysed in turn, applying codes to each person’s file to capture all their experiences and views. With each subsequent participant, quotations were added to existing codes, descriptors/headings were refined/extended, or additional ones generated. A summary of each person’s experiences was created, alongside several working documents that concentrated on key themes: experiences of Blue Cross support (accessing and receiving), type and impact of financial difficulties, challenges of pet ownership (the stressors), and (the main focus here) reluctance to seek support. These included tables of illustrative quotations to enable clear comparison across cases (Braun & Clarke 2006). These were reviewed by the second author (JW) to ensure the integrity of the analysis process.

Close examination of stories enabled identification of common themes and language that related to ways in which participants appeared to be “claim[ing] or avoid[ing] identities” (Wong & Breheny 2021; p 2644), thereby reflecting broader cultural rules or norms. Therefore, these instances were coded as ‘underlying constructs’ and returned to separately. In-depth analysis revealed shared narratives regarding what it means to be a good person – as a pet owner, a member of a pet-owning community, and a moral citizen, as opposed to someone who is not. Bruner (1991) argued that people seek coherence through narratives, especially when they perceive a discrepancy between preferred selves and experiences or between a personal identity and cultural/societal expectations for identity. This was evident among our participants when they found themselves in circumstances beyond their choosing, when they often referred to negative emotions as well as actions they would take in the future to ‘put things right’ (see *Results*).

### Ethical considerations

Our study received ethical approval from the Clinical and Health Psychology Ethics Committee at the University of Edinburgh (CLPS245). We did not ask explicit questions about personal finances or probe particularly difficult experiences. However, participants were often very forthcoming, and on occasion showed signs of emotional distress. The researcher remained responsive to participants and at any point where people seemed upset or were struggling to find the words, she acknowledged the difficult experience that they had had and reminded them that they could stop at any time. At the end of the interview, the participant was thanked for sharing their experiences and offered the opportunity to ask any questions. A debrief email was also sent with details of supportive organisations (Blue Cross, Samaritans, NHS 24, and the Citizens Advice Bureau).

## Results

### The experience of initially accessing support

The majority of participants talked about the process of accessing support being largely unproblematic, and this was often attributed to the welcoming and friendly nature of staff they initially encountered. However, some people hinted at the discomfort they had making that first move. Richard (from London), for example, described feeling *“slightly intimidated by going for the first time, because my circumstances changed completely, you know. All my life changed you know, since I started with the health issues… It’s like you go inside and you know everyone feels like, well I don’t want to say on benefits, you know.”* Suzy (from Grimsby) described feeling very emotional at the kindness of others, having never had to seek help before:

*“When we were in* [previous home town] *we went to one food bank to begin with and we, the lady at the DSS gave us this list and for ages we put off going and* [sigh] *when we went, I can remember the very first time we went, I was in bits. It was just how kind people are. You know, but I think a lot of it that stopped us, I suppose was. I don’t know if we, we probably did feel a bit too proud didn’t we, because we had what we had still, we wasn’t like destitute.”*

Wondering what people will be thinking of you, and not wanting people to know your personal situation were influential. However, the strong assertion made by several people that the animal should come first, and a more ‘matter of fact’ attitude recognising it is important to ask for help when you need it, explains why most of our participants had not held back when they needed support. Lisa (from the Midlands) stated, *“I kind of look at it as if I’ve got a welfare concern, the horse has got to come first. My emotions don’t come into it.”* Christine (from Grimbsy) similarly drew attention to putting your own feelings aside and being honest about recognising when you need help. However, her comments allude to a certain degree of difficulty when she highlights being “strong”: *“I would never be in that position where I wouldn’t ask for help. I think that some, if you’re quite a strong person then you will ask anyway. If you need help you need help.”*

### Participants’ reflections upon what might stop someone reaching out for support

#### Pride, shame and embarrassment

“Being too proud”, as Suzy mentioned above, was often cited as the reason why people might not seek support, though this was often with an air of annoyance, as Martin’s (from London) comments below exemplify:

*“I think the same thing applies to food banks for themselves. I mean, if certain people wouldn’t do that, they’d prefer to suffer in silence and I don’t understand that personally, because if there is a resource out there, why not use it? Erm, first thing I would say to them is it’s a resource for, not for you, it’s for your dog. You know if it’s suffering in any which way that you need a vet, you should think about the dog, not yourself and your pride.”*

Others, however, drew attention to deeper more disturbing feelings than ‘pride’ implies. They highlighted a fear of being judged, assumptions being made about what kind of person you are or how you treat your animal, and feeling ashamed about having to ask for help. Jean (from London) explained clearly how society and the media exacerbates these feelings:

*“It’s embarrassing. It’s degrading but it’s not, but it is, because the media and the government have put out such a rhetoric about, oh so and so’s on benefits and they’ve got a flat screen TV and an iPhone, and you know they’re smoking and they’re drinking and we all get targeted with that a bit and the media…. stirs it up!… Obviously, people will worry you know oh am I not looking after my dog properly or will they make a judgment on me? It’s all about judgment and people pre-judging people.”*

Feeling ashamed to ask for help was often associated with perceived failure or inadequacy and a concomitant feeling of guilt, as Alison (from southern England) explained, *“I think people are embarrassed by it. They feel they’re failing.”* Even among our help-seeking participants, there was sometimes open admission that they had taken on an animal without full knowledge of what that would entail and whether they could cope. Accordingly, they subsequently felt bad about the decisions or actions they had taken and the knock-on effect of needing support that was unanticipated. Gemma (from London) was very honest about her experiences and was taking her responsibility very seriously, describing the sense of guilt she felt:

*“I can see some people just feeling, I guess embarrassed or ashamed that they have to ask for help and to be on benefits and ask for help is err, I think, when we first used the service to get him neutered, we did, but me and my housemate felt quite guilty that you know we were getting it for free… It was really good and we did really need it and we couldn’t afford, like we should have thought through really having a pet, thought everything through, but we didn’t think about it, but obviously we were in the situation where we didn’t have to give him up or, you know, it wasn’t at that stage and we didn’t want to give him up as he is… you know he’s a bit of a problem, someone else might have just got rid of him again, erm other people might have just wanted to breed him. That was a big part of getting him done.”*

Associated with this, there was acknowledgement of the potential repercussions of seeking support for your pet; that perhaps the charity would suggest rehoming. Returning to the notion of shame though, several participants highlighted the stigma of being on benefits or receiving charitable support. Patricia (from the Midlands) took this a step further, explicitly highlighting a broader cultural narrative around poverty:

*“I think there’s still a bit of a shame sort of based thing about being in poverty as though people assume that it’s your fault. You haven’t worked hard enough. You haven’t tried hard enough to get a job. You’re lazy. And I think maybe that will change as more people find themselves in that situation, because it’s like, well, it could happen to anybody. So I think it is a lot of shame, a lot of feeling like they could have tried harder, that it’s their own fault they’re in that mess. I think that’s reinforced, isn’t it by other people and stuff. I still have friends who say things like ‘oh I won’t claim benefits’ or ‘I won’t claim this’. And I’m like, well you paid for it with your National Insurance. It’s not actually charity, you know. And I think the same thing with food banks and stuff, you know the community has provided it for you. If you leave it there, you’re actually rejecting the help and it will just go into landfill so why not take it?”*

#### Wanting to manage independently and denial

Several participants felt some people may not ask for help because they think they can, or *should* be able to, manage by themselves. Alison (from southern England) said, *“I think people feel that **they** should* [be the ones who] *manage their dog”*, while Joyce (from Grimsby) described how she *“felt bad having to rely on other people to sort things out for me at the time, you know.”* Joyce was struggling with her hearing, so she relied upon her daughters to liaise with the veterinary staff when her dog was very unwell. She really appreciated staff members’ efforts to help her by writing things down and removing face masks so she was able to lip-read. However, she did not like, in her words, putting people out in this way. Linked to the sense of pride discussed earlier, our participants also described how some people might not even acknowledge that there is a problem. Suggesting there may even be a cultural dimension to this, Lisa (from the Midlands), explained:

*“I can’t talk on behalf of anybody else, but I do think it’s maybe fear of judgment, and maybe not wanting to sort of admit there’s a problem, wanting to try and deal with it yourself like, I think with Brits especially, there’s a sense of pride isn’t there? Not wanting to ask for help in any areas of life.”*

#### Financial implications

The cost of veterinary care was proffered as a key reason why people do not seek support for their pets, as David (from London) explained, *“I think they’re frightened of getting a big fat bill, mainly. I mean, I try and prevent any health problems as much as I can.”* This was anathema to most of our participants who would clearly have done whatever was necessary to ensure their pets received the care they needed. Michael (from London) exemplified this when asked why people might be reluctant to come forward:

*“I dunno, financial cost I guess would be one. I don’t know really erm, you would think when it comes to your animal, you’ll do anything that you have to do, but you know. All you have to do is pick up the phone or whatever, and just Google whatever it is you need and an answer should be there so I don’t see any reason why it should stop people nowadays.”*

Similarly perplexed, Ann (from Grimsby) was horrified that an animal in her neighbourhood was clearly suffering and the owner apparently doing nothing about it. However, this led her to reflect on the situation for people who, unlike her, were not able to receive financial support:

*“I live in a lovely area, but there’s a cat that’s roamed around now for two years with half its tail missing. But it drips blood… and you know after two years, the tail hasn’t healed and I think, why on earth didn’t she just take it to the vet and get the tail, the rest of the tail just cut off or something…. These are the people. They’re not registered at the Blue Cross to get the help, and they’ve got to pay the full vet, private vet fee.”*

A small number of our participants also described other reasons why people might not seek support. These included not knowing there is support available, not realising they are eligible, not having the capacity to look after the animal (due to mental health/drink/drug problems), or simply not caring, as Sandra (from London) described:

*“Some people like the idea of a pet, but you know, I mean, one of my many friends… she’s got a very well paid job, and she’s got a cat that she absolutely loves. And I remember we would just pop into Tesco or Sainsbury’s where she’s getting what I would call shit cat food… She was also worried that there was something wrong with her cat, so I said well take them to the vet, ‘well it’s so expensive’. But this is somebody who has got loads of money, and yet, if I had any concern whatsoever you know I’d be at the vets however much it costs.”*

Janet (Grimsby) compared pets with children, emphasising how people’s capacity to look after their dependents can be impaired: *“I think with people with drugs and alcohol… although they love their animals, they don’t often, don’t really recognise the needs, well it’s like that with children either. They don’t actually recognise the needs of their dependents should we say, very sadly.”* Two participants also highlighted people who ‘fall through the gaps’, those who do not have the required ‘paperwork’, those who are homeless and have pets, and people (as Ann pointed out previously) who are employed and not on benefits, but are struggling financially. ‘Where do these people go?’ was the question.

### Participants’ own reluctance to reach out for support

Outside of questions pertaining to other people’s reluctance to seek support, there were instances in the interviews where our participants mentioned times when they felt bad receiving support or were not currently asking for help in spite of an apparent need. Guilt, feeling ashamed you cannot handle something on your own, or are the source of the problem with a pet, and fear of financial implications were all involved.

Not wanting to ask too much of a charitable organisation was evident among our participants. People were hugely appreciative of the support and kindness they had received, but some felt a sense of guilt about taking up the charity’s time and resources, and not being able to pay much towards their pet’s care/treatment. Like Dawn (from London) below, they were keen to repay what they felt they owed when they were in a position to do so.

*“I think I felt guilty because I know how bad resources are and I know at some stage I will, because I am on Universal Credit, I haven’t got any income coming in, but I’m in a ridiculously lucky place that I own my property. I’m not claiming housing, though I feel really bad because I know in the in the near future when I sell, which won’t be that long, I keep thinking then I’ll repay and give a donation… I mean there’s nothing I wouldn’t do for the animals but it’s just been, that little help, no that big help now, has been massive, so I suppose I feel guilty, but I know that I’m gonna repay what I feel I owe, because there’s other people and they need so much help.”*

Dawn also admitted, somewhat reluctantly, that she felt bad for doubting the standard of care her animals might receive given the care/treatment was free. She had wondered if she should sell her house in order to ensure the highest quality care for her pet but had subsequently realised that Blue Cross had been incredible.

In Libby’s (from London) case, her family decided to get another dog after the death of their first one. Before they had children, they had fostered dogs, so she clearly felt experienced and had no concerns. However, their new dog, acquired just before lockdown, was exhibiting very difficult behaviours, especially around strangers (people and other dogs). She had paid for a series of sessions with a behaviourist amounting to around £800, but these had been unable to resolve the issue. She admitted feeling *“so ashamed about how bad he is”* and hadn’t gone back to the breeder to ask for advice, because she was blaming herself for the problem: *“I feel really embarrassed that it’s obviously something I’ve done”.* Echoing the issue highlighted above about wanting to manage independently, Libby explained that when their niece expressed an interest in taking the dog, her husband said *“I’m not like a quitter… I feel like I need to get him better”.*

With respect to financial concerns, many participants talked about sacrifices they made in order to give their pet the best care (see below), but only Patricia (from the Midlands) admitted to actually ‘cutting corners’:

*“I mean, I must admit I cut corners with things like flea treatments. I don’t have them as regularly as the vet suggests. I tend to sort of like watch them and see, and if they don’t look like they’ve got fleas then I leave well alone ‘cause it’s money I don’t want to spend. And I do keep all the vaccinations up. That’s sometimes a bit of an issue, but I do it… I think as well, I haven’t had that experience yet, but I think you know, as long as they weren’t in a lot of distress, I might be tempted not to go and see a vet as often because you know if there’s something that’s a bit of a niggle and you think I don’t think they’re gonna die of this, you know, just not go, because it’s £30 before you even start, even before they even look at the animal and it’s like oh gosh no.”*

Other people who had been on a low income long term also mentioned the stress of being on charity waiting lists for their animal to be neutered or vaccinated (due to significant demand and perhaps supply issues), as they could not afford to go privately.

### Participants’ recommendations for improvements to services

Discretion was a theme that underpinned recommendations for changes to systems and practices. Keenly aware of how others, and society as a whole, make judgement calls with respect to those who are struggling or not displaying behaviours that are valued, participants described the need for services to be welcoming and non-judgmental, and sensitive to the need for respect and privacy, recognising that most people do not want to be in the position where they need to seek support. Christine (from Grimsby), for example, described how the lack of privacy at the front desk had made her feel uncomfortable, let alone others who are more embarrassed about being there:

*“A couple of times when I’ve been in, the receptionist question things. It’s very open in there and there’s lots of people stood around and when they’re asking questions and saying let me have a look at your claim, I want to make sure, I need to have a look. I don’t think that’s great sometimes. Some people won’t be bothered, yeah but I think some people will be a bit more reserved. I wouldn’t like that because I’m a bit more reserved… and it can be quite embarrassing for some people I would imagine.”*

This also applies to the way services are signposted, with the suggestion from a few individuals (Dawn from London is provided as an example below) that these are somehow reframed so that they are viewed as a ‘vital service’ or akin to the NHS or Citizens’ Advice, to distance the provision from stigma associated with being on benefits, in poverty or receiving charitable support, and any negativity related to not being able to cope or manage on your own:

*“Somehow I can think of it more as a health service, almost? Yeah, and maybe charities like Mind should have a thing like if you have companion animals maybe think of like the relationship and they’re here for you and here is where you go, you know, sort of more like* [sigh] *it’s not like, oh God, it’s a charity case. It’s like, it’s a service to help, because people don’t feel bad about going to Citizens Advice, but that’s a charity and that’s free.”*

### Wider narratives at play

Three shared narratives came through strongly in the interviews that show how wider societal attitudes and values shape identities and behaviours. These related to ‘giving back’, ‘sacrifice’ and the notion of ‘equity’.

#### Participants need to ‘give back’

‘Giving back’ denotes a wider cultural belief that you give back what you take out, the moral obligation to return the favour. The desire to ‘give back’ was evident in many participants’ narratives, usually with respect to the donation given to Blue Cross for pet treatment, but people also talked about taking on rescue animals as a form of ‘giving back’, donating things for animal welfare charities to sell in their shops, taking part in the charity lottery, or as Suzy (from Grimbsy) highlighted, volunteering their time, *“We actually went in to the animal hospital and we asked about volunteering for probably a couple of days to work in the shop. We’re not doing anything so we just thought why can’t we do that, give a little bit back.”* All participants registered with Blue Cross were entitled to free or low-cost treatment, but in the case of the former, were always invited to give a donation following treatment. The amount or frequency of giving a donation was clearly uppermost in participants’ minds, alongside a strong desire to do the right thing. As Jean (from London) pointed out, *“You’ve got to try and give as much as you can when you do take a service. That is important, but you shouldn’t be judged if you can’t give the whole amount.”*

Emphasising how they did not just give the minimum or a small proportion of the cost if you were able to pay more, they also recognised that there may be times when you are not able to. Christine (from Grimsby), for example, explained, *“When I used them last, they did an operation on my little Chihuahua that I’ve got now, but I paid the amount what it costs for the operation anyway because I just think if you can then you should, erm, but at times I’ve not been able to.”* Those who could not afford to pay anything at the moment, like Gemma (from London), were clear to point out that they would pay it back when they could: *“I think I said the last time, as you get asked for a donation normally after a visit which is really, I haven’t been able to donate, but I did say last time once I’m working for a while and have spare money, I’m gonna donate as I do want to give back.”* Similarly, David (from London) said he always pays the full amount. He was one of the few people who described pre-empting problems, having had a traumatic experience with his ex-partner’s pet in the past, *“I mean, I try and prevent any health problems as much as I can, but they say the whole donation I think is £25 or £27, do you want to pay a fraction of that? I just pay all of it.”* This theme clearly highlights the importance, in our participants’ eyes, of being a responsible pet owner and good citizen. David, in particular, explained that in spite of being eligible for low-cost veterinary care and only needing to pay a proportion by donation, he was able to pay the full amount because he prioritised his pet above anything for himself, the theme to which we now turn.

#### Making sacrifices to ensure your animal is well cared for

The majority of participants demonstrated the strength of their bond with their pet and a commitment to them through the language of sacrifice, putting the animals’ needs first, always above their own. David described his pet as *“my one big luxury really. So when people gripe about contributing to a charity, I think well just drink and smoke less. Above my luxuries, I would go without. He comes first, but not everyone looks at it like that* [laughter]*.”* Many participants (like Helen from Grimsby) described how changes in circumstances, often combined with the cost-of-living crisis, meant they had to economise, *“I mean, you know my husband’s retired. I’m retired now and we just can’t afford, yeah you know things and you’ve just got to pull your things but I mean I love my dogs so wherever I have to pay for them, I’d rather go without myself than have them not looked after.”* This ‘pulling back’ rarely affected the pet’s care, because they ‘came first’, but would affect the owner. Sandra (from London) explained this in relation to food, *“I’m bonkers about them you know. My dogs actually eat better than, well they eat better than me, but they eat better than a lot of people.”* Joyce (from Grimsby) also explained how going without yourself to look after your pet operated in the same way you would sacrifice things for the sake of your child(ren), *“I’ll go without to pay her bills you know what I mean. It doesn’t bother me. She comes first. It was like with my kids, they came first.”* This was the case, even when the animal’s behaviour had caused a lot of distress, as in Gemma’s (from London) situation:

*“I would have preferred to rescue an animal, but err, it just is what it is and erm, you know, since finding his issues, I’m not gonna give up on him like you know the first people did, as he’s, you know, I’ve worked with him for a couple of years now and he’s gotten so much better than he was… I always make sure for money, money wise that he’s taken care of before me as he can’t take care of himself.”*

#### Comparing with others and the significance of equity

Importantly, with respect to understanding a reluctance to seek support, there was a common tendency for participants to: (a) question whether others should be eligible for financial support for their pets; and (b) compare themselves and the donations they made with others who do not appear to be doing the same. The result being some resentment (expressed below by Sandra from London), towards those who appear to be ‘taking advantage’ and stretching the system of support:

*“I mean there are people that you know screw the system whoever they are ‘cause they’re that sort of people. And I think sometimes these people who are just throwing coppers on the counter at Blue Cross, I just think mmm you know ‘cause you clearly, you’re dressed well, you know, you’re clearly not going without yourself… I don’t think they work for a living quite honestly, and they’re smoking and they’ve got a fancy phone… It’s not that they can’t afford it, they just don’t. And I’m not like that, I’m generous with the donation that I give.”*

When the researcher asked “*If you could offer some advice on that, you know in terms of what Blue Cross might do to improve things or target the people who are really struggling?*”, Christine (from Grimsby) responded:

*“Well, I don’t always think it’s about people who are struggling…because I think some of those people take advantage if I’m being honest… Even though I get, I can use free treatment and I can do it contribution based, when I’m in there I see a lot of people who come in with family members and you can’t prove that that dog’s yours. I think that now they do check the chip and everything, but I think even if someone, they had to pay something every time they went instead of it being donation based, I think people would be a bit more loath to go.”*

Joyce (from Grimsby) similarly mentioned, and distanced herself from, those ‘taking advantage’:

*“People can take advantage you know, because it’s a donation only. A lot of people do take advantage of it, and I think it’s all wrong myself. I mean if I could afford it, I’d pay you know the full price, ‘cause I think it’s like other medications that Polly was on. It’s like I paid the majority of it, not the full amount but I paid the majority of what it was.”*

At the same time as these comparisons were being made, with a sense of unfairness relating to the sacrifices they have to make, there was also recognition in a few interviews of the need to not make judgements about others. Ann (from Grimsby), for example, explained how *“sometimes you sit in the waiting room and you look at some of the pets there and you’re thinking my goodness, if you, you shouldn’t, but I sort of think you’ve got this luxury thoroughbred dog sitting there, why are you needing, using these services? And I know you shouldn’t because my circumstances changed as well.”* This contradiction, also evident in Suzy’s (from Grimsby) quotation below, highlights that it is part of human nature to make comparisons (especially perhaps with regard to fairness), even when we know, as a good citizen and caring person, that we should not pre-judge:

*“I don’t judge, I’m not judging, you know, just the way they are, the way they look, and it’s just really quite sad to see that although they’re trying their best for their pets as well so err, I don’t want to be horrible. I do think there’s a few people that could abuse the system, because Liam and I don’t drink, we don’t smoke, all of our time goes with Gilly. We hardly go out… we can’t really afford to, so we prioritise things. There’s a lot of people there that don’t seem to prioritise you know, they’re still, they can’t help it, it’s how they are. They’ve probably got a drug habit. They’ve got an alcohol problem. But they talk and they don’t talk quietly with each other and you can hear, and you think well if you can afford to be in the pub every day, or to buy like ten cans of beer or cider or whatever it is you’re buying, why aren’t you prioritising your pet? And it feels like you know, they don’t have to because they’ve got their* [charitable support].”

Although there was recognition that people fall on hard times and should be helped when they really need it, there were clear concerns that a lot of people ‘take advantage’ (never making any donation at all, not prioritising their pet, or making any sacrifice on their part, and putting undue strain on the charity). There was a strong sense of the current situation being unfair, with some people making suggestions as to how this might be better managed (e.g. a basic monthly payment made by all recipients of support). In essence, this narrative links to being a good moral citizen, both within the pet-owning community and more broadly – thinking about other pet owners and people in need.

## Discussion

The ‘ideological’ narratives (Haidt et al. 2009) uncovered in this study highlight the identities with which we wish (or, more importantly, do not wish) to be associated, thus providing a strong indication of the reasons why people might not seek support even when they are eligible and really need it. The language of ‘sacrifice’, and ‘giving back’ reinforce the idea that a good person is someone who does the right thing and is trying hard. They put others’ needs first, they contribute and are competent. They are good pet owners and members of society. They are not lazy, failing or irresponsible, characteristics often ascribed to those who find themselves struggling (Matheson & Pranschke 2022; Ivancic & Dooling 2023).

People generally do not want to take from the system without giving something back, and undoubtedly never want to be considered someone who ‘takes advantage’ or ‘screws the system’. But what if you find yourself in the unenviable position of not being able to ‘pay it back’, there is nothing left to ‘sacrifice’, or you are unable to take control of the situation you find yourself in? What if you feel you will never be in this privileged position? With specific reference to food assistance programmes in the US, Ivancic and Dooling (2023; pp 309–310) argue that shame (also understood as internalised stigma; Clement et al. 2015) and exposure are built into their very structure, making invisibility an attractive alternative. Individuals, they contend, “must repeatedly ‘out’ their status to strangers”. This creates what they term ‘entangled shame’ – “an embodied experience of profound embarrassment” and “the communicative process through which people convey food-seeking [or any other form of support seeking] as shameful”. People feel entangled shame because the “system strips them of their dignity and societal actors uphold demeaning discourses and structures undergirding the system”. This was evident among some of our participants who spoke about not wanting others to know their personal business, and being subject to cultural stereotypes, highlighting what has been described as a media and political discourse of ‘scrounging’ (Baumberg *et al.* 2021) or the ‘undeserved poor’ (Shildrick & MacDonald 2013).

Baumberg et al. (2021) suggest that there have been improvements in the way claimants are treated by the Department for Work and Pensions (DWP) in the UK (i.e. treating with dignity and consistently speaking respectfully about claimants), especially during COVID-19, that ought to reduce benefits stigma. Media portrayals might also have altered in light of societal changes. However, there is clearly a legacy that is likely to take considerable time and consistent effort to eliminate. People feel bad enough about themselves if they fall on hard times (Inglis et al. 2023). If support services are not sufficiently sensitive to this, they may alienate the people they most wish to help. It is easy to see how a negative experience when at a low point might inhibit people from reaching out again. They already feel guilty and are taking responsibility for their own perceived failures by trying to cope alone and not burden society. Taking that first step to seek help requires people to admit to a lack of control in their lives, which is hard (Nuske & Hing 2013).

Messages and values that permeate society and result in entangled shame need to be tackled head on and explicitly challenged when advertising available support and delivering services. Our participants, who had sought support, had found their experience with Blue Cross, and food banks, to be full of compassion and care, and no judgment. This may well be due to Blue Cross’ (personal communication 2023) attention to the messages they are sending out, using the COM-B model (West & Michie 2020), a framework for understanding human behaviour change, to shape their communications. Sharing meaningful testimonials that actively address any of the common concerns people have may well be another useful strategy.

It is entirely possible, as a result of rising costs, that far more people than ever before are now putting their own feelings and any thoughts of potential implications aside and reaching out for financial help with pets, the language of sacrifice revealing the strength of the human-animal bond and the need to look after their animal companion well (Wensley 2008; Walsh 2009; Sable 2013). Some of the rhetoric, stereotypes and discourses around poverty may also have been disturbed in light of widespread recognition, as Patricia highlighted, that a dramatic change in circumstances “could happen to anyone”. Notwithstanding the increased demands on charities to increase their provision of low-cost veterinary care and pet food (Blue Cross 2022, 2023, personal communication 2023; Scottish SPCA 2023), there are still people who feel they have no choice but to rehome or even abandon their animal.

It is also important to recognise that people may not be struggling to breaking point, but nonetheless find it difficult to balance financial demands. In these cases, as some of our participants highlighted, they may feel the need to ‘cut corners’ with respect to pet care (see also Citizens Advice Scotland 2023). If people are not eligible through means-testing to receive financial support (or do not think they are eligible), they may feel they have no choice. This is one area in which animal welfare charities could work together in a similar way to the Pet Education Partnership (PEP). This website provides comprehensive pet education resources and is the result of a collaboration between eight leading charities in the UK. A ‘what help is available where’ website, representing a ‘one stop shop’ for people who need support with their pets, could be extremely helpful in encouraging people to approach the right organisations, especially if the language used acknowledged both the sacrifices people make for their pets and their feeling that they need to ‘give back’ (volunteering is one such avenue that could be emphasised).

The increasing cost of veterinary care was highlighted as a key concern, with some of our participants being unable to provide their pet with the care they needed if the charity was unable to supply it. A review into veterinary services for household pets has recently been launched by the Competition and Markets Authority (CMA) (Gov.uk 2023b) “amid concerns that pet owners might not be getting a good deal or receiving the information they need to make good choices”. This is clearly a widespread issue that is undoubtedly contributing to the increased demand for low-cost veterinary care that now far exceeds the capacity of animal welfare charities (Blue Cross 2023, personal communication 2023). Provision of behavioural support for pets, dogs especially, also appears limited and expensive. Challenging behaviours are a prime reason for relinquishment (Coe et al. 2014; Buller & Ballantyne 2020; Jensen et al. 2020) and another source of embarrassment and shame for owners, suggesting that greater provision in multiple forms, and early intervention, would reap rewards in terms of keeping people and pets together.

A ‘reframing’ of services that support ‘people and their pets’ (i.e. signalling discretely their purpose to help people through difficult times) was suggested by some of our participants to help challenge any negative associations with being a ‘charity case’. They felt it would be helpful if these were articulated in the following terms: an NHS service for pets, or a ‘vital service’. Food banks that advertise with a focus on preventing surplus food going to landfill have helped to draw people in (some people swapping food where they feel they want to give back). There have also been some creative collaborations between organisations that help to target people who may need support but are not seeking it. One of our participants (Dawn) highlighted the Kleenex/Mind collaboration (Mind 2023), where tissue boxes were designed in different colours with everyday reminders to take time to support your mental health. It may be that animal welfare charities could build some effective partnerships to similar effect. The donation from Kleenex, for example, will fund up to 25,000 calls to Mind’s Infoline. However, it is important to acknowledge the increasing demand on animal welfare charities to somehow compensate for lack of financial support in other arenas. They can only do so much with limited funds.

### Limitations

Our sample represents people who have sought support, but also those willing to take part in our research and talk about their experiences. Our participants were also a group of ‘pet lovers’, enthusiasts about, and advocates for, animals, often having rescued animals to give them a better life and volunteering their own time to help animals. Their experiences of struggling had not impacted on the pet in the majority of cases, as they put the pet first (see also Arluke 2021) and had managed to keep caring for them largely because of the help they were receiving from Blue Cross. It is possible that others do not view their pets in the same way as our animal lovers, and financial struggles may well affect the bond people have with their pets, particularly where mental health has been compromised (Muldoon & Williams 2024). These are areas where research is required. However, reaching those who perhaps do not have the same positive relationships with their pets, those who are facing significant challenges across all aspects of their lives, or those who feel they are not eligible or entitled to support, is incredibly difficult. Very few people want to admit to doing things that society frowns upon (even if they feel they have no choice), so a creative approach is undoubtedly needed to reach those who are essentially ‘invisible’ due to avoidance of stigmatisation and shame (Shildrick & MacDonald 2013; Inglis et al. 2023) or an insistence that they must manage on their own.

### Animal welfare implications

The implications for pet animals when their owners do not seek necessary support are clear. They may not receive the medical, nutritional and physical care they need, any behavioural issues may escalate or, in the worse-case scenario, animals may be abandoned. In cases where the pet animal means the world to their owner, people make enormous sacrifices themselves so that their pet does not go without (see Arluke 2021; Muldoon & Williams 2024). This means there are implications not only for animal, but also human, health and well-being. If support is not in place for people as well as their pets, any problems are likely to reoccur or get worse. Thus, there are strong arguments for pet animals to be considered in the process of existing social support and social security provision (Muldoon & Williams, in press).

At present in the UK, pets are not factored in to Benefits Office assessments of people’s financial situation, their dependents and network of support. It is not a formal element of the support and advice given by the Citizens’ Advice Bureaux, nor are pets mentioned on Government websites that provide information on support for those in receipt of means-tested benefits. Animal welfare organisations that provide reduced cost/free veterinary care and other services need to be clearly, but discretely, signposted. Social work practice would also benefit from formal consideration of pets in their assessments of need, especially, it seems, where older people are concerned, as social workers recognise decreased capacities for self-care (Walters et al. 2001; Howse et al. 2004; Bibbo et al. 2022).

Animal welfare organisations welcome referrals to support people when an animal may be suffering from neglect, unintentional or otherwise. It is vital that these organisations send the right messages to support those in need who are fearful or reluctant to reach out. While media portrayal of some of these organisations is changing, coverage in advertisements often focus on intentional neglect and harm of animals, extreme cases, and human abuse. This is likely to colour views on what will happen; the implications of reaching out when people know their animal is not in the best of health or feel they have let their animal down. The issues identified here that are likely to prevent people seeking help also need to be addressed head on. It seems necessary for charities to address many participants’ concerns about the fairness of the system in terms of payment (specifically voluntary donations) for services. There is a strong perception that some people abuse or over-use the system, leaving charities with no capacity to reach those who may not wish others to carry their burden but legitimately need support to cope. The extent to which this is true is undoubtedly difficult to pinpoint, but it seems important that the issue of equity is addressed to help users of services understand that the charity system they are accessing is fair.

## Conclusion

Not everyone who is eligible for, or in need of support seeks it. This study explored the reasons why this might be the case when people are struggling to look after their pets. Our participants had sought help when they became aware that they were eligible. Their experiences of initially reaching out, and reflections on why others might not do the same, shed light on the issues associated with reluctance (e.g. pride, embarrassment, guilt, wanting to manage alone, concerns about privacy). Yet, it is the shared narratives that were evident in their stories, of ‘sacrifice’, ‘giving back’, and ‘equity’, that really get to the heart of the matter, identifying what it means to be a pet lover, a responsible pet owner, and a moral and giving citizen (within the pet-owning community and beyond). They signal the identities and behaviours that are (and are not) of value in our society and that contribute to stigma. A single source of information showing pet owners all the sources of support that are available would undoubtedly encourage people to come forward. However, support that is available also needs to be discretely advertised and delivered, ‘reframed’ if necessary, in order to diminish any negative associations and implications. Opportunities for people to ‘give back’ in different ways (volunteering/donating items for shops/paying back later when in a position to do so) also appears important. Otherwise, we will continue to see many animals not receiving the care they need.

## Supporting information

Muldoon and Williams supplementary material Muldoon and Williams supplementary material

## Supplementary material

The supplementary material for this article can be found at http://doi.org/10.1017/awf.2024.19.
