# Supplementary material for: Why are some people in the UK reluctant to seek support for their pets?
Source: Anim Welf. 2024 May 3;33:e25. doi: 10.1017/awf.2024.19 (PMC11076913; doi:10.1017/awf.2024.19)
Supplement: Muldoon and Williams supplementary material [file S0962728624000198sup001.docx]

Supplementary material: The interview guide/prompt

| **Background** | Welcome participant, brief intro and chat, asking age and where they lived before starting the audio-recording.  Ensure the participant understands the research and if they have any questions. |
| --- | --- |
| **Accessing Blue Cross support: reasons and experiences** | Confirm which Blue Cross services were accessed and for which type of pet/s, and why they initially went to this organisation.  Identify the type of challenges/issues experienced before they accessed services (i.e., health/behavioural issues/financial).  Identify if there was a tipping point that led the participant to seek help from Blue Cross.  Establish initial impressions of Blue Cross services and the processes involved.  Ascertain how easy/difficult it was to seek support (to identify potential barriers). |
| **Type of support received and the positives and negatives** | Pinpoint the type of support received and how it helped or affected the participant and their pet.  Identify key qualities of Blue Cross as an organisation, and the impact of receiving support.  Identify any problems in the process of accessing or receiving support from Blue Cross.  Ascertain if they remember why they accessed Blue Cross services rather than any other providers/awareness of other organisations. |
| **Barriers to accessing services** | Establish what helped the participant to access services.  Identify participant’s views on why some people might be reluctant to access services.  Ascertain if they have advice they would give to someone who is reluctant but really needs support.  Pinpoint any changes Blue Cross could make to improve their services or make them more accessible in order to reach people who are not currently reaching out. |
| **Thanks and debrief** | Thank participant for taking part and acknowledge any difficult times they have experienced.  Discuss what will happen next (sent an email with list of organisations providing support, a key contact at Blue Cross and a link to our webpage).  Ask for permission to send the Amazon voucher to their home address and procedure for doing so. |
